# Second-hand smoke exposure and the factors associated with avoidance behavior among the mothers of pre-school children: a school-based cross-sectional study

**DOI:** 10.1186/1471-2458-10-606

**Published:** 2010-10-14

**Authors:** Pi-Li Lin, Hsiao-Ling Huang, Kuei-Yun Lu, Ted Chen, Wei-Ting Lin, Chien-Hung Lee, Hsiang-Ming Hsu

**Affiliations:** 1 Graduate Institute of Medical Science, Tzu Chi University, 701, Zhongyang Road, Sec. 3, Hualien, 97004, Taiwan; 2 Department of Nursing, Meiho University, 23, Pingguang Rd., Neipu, Pingtung, 91202, Taiwan; 3 Department of Oral Hygiene, College of Dental Medicine, Kaohsiung Medical University, 100 Shih-Chuan 1st Road, Kaohsiung, 80708, Taiwan; 4 School of Nursing, Fooyin University, 151, Chinhsueh Rd., Ta-liao, Kaohsiung, 831, Taiwan; 5 Department of Community Health Science, School of Public Health and Tropical Medicine, Tulane University, New Orleans, LA 70112, USA; 6 Department of Public Health, College of Health Science, Kaohsiung Medical University, Kaohsiung, 80708, Taiwan; 7 Department of Public Health, Tzu Chi University, 701, Zhongyang Road, Sec.3, Hualien, 97004, Taiwan

## Abstract

**Background:**

Second-hand Smoke (SHS) exposure is a significant public health problem that may be responsible for serious health hazards for child. This study aimed to examine the exposure status of SHS and the factors associated with SHS avoidance behavior among the mothers of pre-school children.

**Methods:**

A cross-sectional study was used to obtain a sample of the mothers of pre-school children (n = 1,020) in 30 registered kindergartens in eastern Taiwan. Overall, 919 (a response rate of 90%) completed the questionnaires. Regression models were used to identify factors with respect to the avoidance behavior of SHS.

**Results:**

The prevalence of exposure to SHS was 70% and 50% for the mothers and their children, respectively. After adjusting for other variables, mothers who were current smokers (β = -0.260, p < 0.001), had spouses who smoked (β = -0.060, p < 0.05), SHS exposure (β = -0.138, p < 0.001), and/or children with exposure to SHS (β = -0.084, p < 0.05) were found to be less likely to avoid SHS, whereas mothers with a high knowledge score about SHS (β = 0.082, p < 0.01), positive attitudes (β = 0.274, p < 0.001) and a high self-efficacy level in regard to the avoidance of SHS (β = 0.397, p < 0.001) were observed to be more likely to avoid SHS. Regression analyses confirmed that the significantly factors associated with the avoidance behavior of SHS were self-efficacy, being a current smoker, and the attitude toward the avoidance of SHS to be that of 55.5% of the total variance explained (p < 0.001).

**Conclusions:**

The high prevalence rate of exposure to SHS for mothers and their children suggests that a well-designed future intervention program should be implemented in regard to pre-school children's mothers in order to prevent these mothers and their children from SHS exposure hazards, more particularly, to strengthen the knowledge base, to enhance self-efficacy and to foster a more positive attitude toward the avoidance of SHS in the mothers.

## Background

Exposure to Second-hand Smoke (SHS), also called Environmental Tobacco Smoke, is a serious health hazard for children and causes substantial morbidity [1], including bronchitis, acute respiratory illness [2], and numerous chronic respiratory diseases [3]. Previous studies have shown that exposure to SHS could result in a stronger risk of incident wheezing or asthma illness among younger children as compared to older children [4,5], and billions more as to the annual loss of life [6]. The cost of SHS exposure was in the billions of dollars in regard to medical expenses during childhood in the U.S.[7].

According to the 2002 Taiwan National Health KAP Interview Survey, the smoking population was 4.89 million, with 50% of males and 5.8% of females found to smoke [8]. Indeed, the higher smoking prevalence among Taiwanese men makes SHS exposure an important risk factor for the exposure of nonsmokers to greater morbidity and mortality in their households. The U.S. Surgeon General has declared that child SHS exposure is not at a risk-free level [9]. In the year 2000, 25.1% of children in the U.S. had been exposed to SHS [10]. In 2004, the Taiwan Children's Health Study found that the prevalence of SHS exposure for Taiwanese children was then at 44.9% [5], or about 20% higher than that found for children in the U.S.

Moreover, the SHS exposure of non-smoking American women appears to be comparatively low, that is, 13.2% [11], whereas the SHS exposure of women in Asia is much more prevalent. A cohort study conducted from 1997 to 2000 in Shanghai, China found that the prevalence rate of SHS exposure for women there was 53.5% [12]. Even more startling, a nation-wide health survey in 2001 found that the prevalence of SHS exposure for women in Taiwan is 58% [13]. These findings unambiguously indicate that immediate action must be taken in order to protect women and their children from SHS exposure, especially in East Asia.

Smoking among fathers is another concern of particular interest [5], as the women and young children who live with them are especially vulnerable to SHS at home. Smoke-free legislation has been effective in protecting both non-smoking adults and children in public places [14-16]. In July 2007, the promote smoking control law, which included the aim of preventing second-hand smoke in public places, was introduced in Taiwan. However, as of 2009 such Taiwanese governmental legislative protection had not been enforceable in private homes, which is still the primary source of SHS exposure for women and children [5,13].

Mothers' avoidance behavior of SHS plays a critical role in the exposure of children to SHS. If mothers do not take adequate precautions to avoid exposure to such SHS hazards, they and their children may be susceptible to a series of negative health effects [17,18]. In Taiwan, mothers are the main caregivers for children, and they have more direct contact with their children at home. Hence, by effectively protecting women from SHS health threats, consequent dangers to the health of their children can also be avoided [19]. Given the relatively low smoking rates among women in East Asia this, coupled with their high prevalence of SHS exposure, it is possible to devise practical and effective approaches to encourage and empower women themselves to reduce their and their children's SHS exposure rates. Yet very little attention has been paid to having a clear understanding the factors associated with a mother's behavior in regard to dealing with SHS hazards. As a result, this study aimed to examine the exposure status of Second-hand Smoke (SHS) and those factors associated with SHS avoidance behaviors among pre-school children's mothers.

## Methods

### Design and participants

Data on the SHS avoidance behavior by the mothers of Taiwanese kindergarten attending/aged children was obtained from the Smoke-free Home Survey Project in eastern Taiwan in 2006. The survey was originally constructed to collect data related to SHS avoidance behavior as well as its potential determinants among schoolchildren and their mothers. Sixty-two of the registered kindergarten schools are located in Hualien County, the largest county in eastern Taiwan, with 10 located in this county's central region, 40 in the northern part, and 12 in the southern part. The participants were invited to take part in this study by means of the issuance of formal letters from the Hualien County Bureau of Health. A total number of 30 kindergarten schools agreed to participate in this study between March and June of 2006. Among these schools is: 5 are located in this county's central region, 19 in the northern part, and 6 in the southern. Non-significant difference was found between mothers of the kindergartens who agree and who disagreed to participate in our study.

A total of 1,020 eligible mothers met the inclusion criteria for this study, and these women received additional information about the study as well as consent forms. Overall, 919 of these mothers (a response rate of 90%) consented to take part in this study and completed the questionnaires sent to them. All of the participants in this survey voluntarily filled out the questionnaires and were assured of retaining their anonymity.

### Procedure

The kindergarten teachers were invited to act as questionnaire facilitators in this study. A one-day training program was provided to all of these facilitators in order to ensure that they could properly explain and administer the questionnaire survey according to standardized study protocol. The survey instruments were constructed based on a conceptual framework that was developed after a comprehensive literature review. They were examined for face and content validity by five local experts in tobacco hazard prevention and public health, as well as by researchers and scholars in order to determine the validity of the questionnaires in terms of accuracy, appropriateness and adequacy. Modifications were made to enhance the content validity of the questionnaire. To assure that the content was well understood by our participants, the questionnaire was piloted in a convenience sample of 68 children's mothers from two kindergartens in Hualien County. Items were revised as needed according to the results of this pilot test. Analysis of the pilot data showed excellent internal consistency.

### Measurement

#### The dependent variable: the avoidance behavior of SHS

An avoidance SHS scale adapted from Martinelli [20] was used to measure the avoidance behavior of SHS among mothers. To assess the participants' behavior toward SHS, the scale used nine diverse SHS exposure situations, for example, in the home and in other places. Examples of the avoidance behavior of SHS included the following general actions: (1) Refusing to enter an environment where SHS is present; (2) Controlling passive exposure by requesting smokers to stop smoking; (3) Attenuating exposure when unable to fully withdraw from SHS; for instance, opening the window to diffuse the smoke in the room. Responses took the form of a five-point Likert type scale where a rating from 1 (none) to 5 (very extensive) was completed. Cumulated scores were summed up at a range of from 9 to 45, with higher scores indicating a better behavior to avoid SHS level. Cronbach's alpha coefficient of this scale was 0.82.

#### Independent variables

*Participant demographics*: these consisted of age, educational level, the monthly income of the household, the smoking status of participant, the smoking status of the spouse ("non-smoker"/"current-smoker"). As in national Behavioral Risk Factor Surveillance System (BRFSS) surveys, a current-smoker was defined as a person who had smoked more than 100 cigarettes in their entire life and who currently smoked on all or some of the days in a month [21].

*SHS exposure*: Participants reported whether or not they were exposed to second-hand smoke for the past 7 days in the home and/or in other places.

*Knowledge of the *SHS *scale*: The following 12-item scale developed by the authors was used to assess the participants' knowledge about the adverse health effects of SHS exposure as well as in regard to ways to avoid SHS: "Second-hand smoke is generated by the side-stream (the burning end) of a cigarette or by the exhaled mainstream (the smoke puffed out by smokers) of cigarettes"; "As long as I do not smoke, long-term exposure to second-hand smoke will not be harmful to my health"; "A smoldering cigarette is more toxic than smoke exhaled by a smoker"; "If not actively smoking, one does not have to worry about damage to one's health from second-hand smoke"; "If one is a current smoker, one's spouse has a higher risk of developing lung cancer"; "If the husband is a current smoker, the wife has a higher risk of developing cervical cancer"; "A lit cigarette burning in an ashtray will not affect the health of people nearby"; "Long-term second-hand smoke exposure during pregnancy contributes to adverse infant health outcomes, including particularly low birth weight and prematurity"; "Long-term second-hand smoke decreases lung function but does not affect the incidence of cardiovascular disease"; "Long-term second-hand smoke contributes to a high incidence of lung cancer in non-smokers"; "Only train and airplane passengers cannot smoke, but car passengers can smoke"; and "Second-hand smoke is a toxic cocktail consisting of carcinogens". Possible responses here included "true," "false" or "unknown" with possible scores ranging from 0 to 12, with higher scores indicating a better degree of knowledge about SHS. In this study, a 0.69 Kuder-Richardson 20 reliability scale level was deemed acceptable.

*Attitudes toward the avoidance of the SHS scale*: Attitudes toward the avoidance of the SHS scale were based on Ajzen's Theory of Planning Behavior [22]. The questionnaire contained a total of six attitude questions and was measured on a five-point Likert scale with ratings from 1 (strongly disagree) to 5 (strongly agree). Cumulative scores were summed up to a range of from 6 to 30, with higher scores reflecting more positive attitudes toward SHS avoidance behavior. Cronbach's alpha coefficient for this scale was 0.89.

*The self-efficacy of avoiding SHS scale*: Items were adapted from Bandura's self-efficacy theory [23], which is used to measure the level of confidence of participants who believed that they could avoid SHS exposure in different situations. The five-point scale's ratings ranged from "extreme confidence" (a score of 5) to "not at all confident" (a score of 1). Cumulative scores were summed up to a range from 6 to 30, with higher scores reflecting a better self-efficacy level of SHS avoidance. Cronbach's alpha coefficient for this scale was 0.82.

### Ethical considerations

This study was reviewed by and later granted permission to be conducted by the Bureau of Health in Hualien County, Taiwan. Each questionnaire was prefaced with a letter that described the purpose, time spent, reward for completion, and as to the voluntary nature of this study, which assured the respondents of the anonymity and confidentiality of their responses.

### Data analysis

SPSS for Windows 15.0 was used for the descriptive and inferential statistical analysis here. Descriptive analyses were carried out to examine the independent and dependent variables. Univariate analysis and Pearson product-moment correlation coefficients examined the relationships observed. The variance inflation factor (VIF) values were less than 10, indicating no multi-collinearity. A Kolmogorv-Smirnov test was performed to test for normality of the distribution (*p *> .05), while a Cook's Distances was performed to indicate if there were no outliers (less than 1). The independent variables were sequentially entered into the regression models to determine the significant variable of a mother's avoidance behavior of SHS.

## Results

### Participants' characteristics

Table 1 summarizes the demographics for the total sample (N = 919) in terms of age, ethnicity, educational level, household monthly income, the smoking status of the study's participants, the smoking status of the participants' spouse, SHS exposure time, SHS exposure location, and the child's SHS exposure time. The average age of the study participants was 33.3 ± 5.0 years, most of which were of the Minna ethnicity (n = 387, 42.4%) while twenty-five percent of the participants were of aboriginal ethnicity (n = 229). Nearly 46.1% (n = 407) of the participants had an educational level of college graduate or higher. The majority of the participants had a monthly household income of 30,000~50,000 New Taiwan dollars. Most of the participants were non-smokers (n = 812, 89.5%) and fifty-two percent of the spouses of the participants were deemed current smokers. More than one-third (n = 317, 38.7%) of the participants reported that they were exposed to SHS 3 hours (or more) per week and their average SHS exposure times were 7.81 ± 18.24 hours. The most common location in regard to exposure to SHS was at home, followed by at work. Of all the participants, 571 (69.7%) and 407 (49.8%) were considered to be either mothers or children exposed to SHS, respectively. The average household SHS exposure time for the study participants' child was 4.07 ± 12.32 hours.

**Table 1:** Descriptive information on individual characteristics in the pre-school child's mothers from 30 kindergarten schools in Hualien County, Taiwan (N = 919)

Characteristic	N	%	95% CI
**Age (y) Mean ± SD**	33.3 ± 5.0		(32.92, 33.60)
20-30	280	30.5	(27, 33)
31-40	568	61.8	(60, 66)
≥40	71	7.7	(5, 9)
**Ethnicity**			
Minna	387	42.4	(40, 47)
Hakka	157	17.2	(15, 20)
Aboriginal	229	25.1	(22, 27)
Other	140	15.4	(13, 18)
**Educational level**			
Junior high school or below	135	15.3	(12, 17)
Senior high school	340	38.5	(35, 41)
College or above	407	46.1	(44, 51)
**Household monthly income (N.T.$)**			
< 30,000	149	17.5	(15, 20)
30,000 - 50,000	277	32.5	(30, 36)
50,000 - 70,000	210	24.6	(22, 27)
70,000 - 90,000	123	14.4	(12, 17)
> 90,000	94	11.0	(9, 13)
**Smoking status**			
Non-smoker	812	89.5	(87, 92)
Current smoker	95	10.5	(8, 13)
**Smoking status of the spouse**			
Non-smoker	424	48.3	(45, 52)
Current smoker	454	51.7	(48, 55)
**SHS exposure time (h/wk) Mean ± SD**	7.81 ± 18.24		(6.59, 9.16)
non	248	30.3	(27, 33)
1-2 hour	254	31.0	(28, 35)
≥3 hours	317	38.7	(35, 42)
SHS exposure location *			
Home	308	33.5	(29, 36)
Work	301	32.5	(31, 37)
Restaurant	248	27.0	(23, 29)
Bus station	235	25.6	(22, 28)
Other	114	12.4	(11, 15)
**Child's SHS exposure time (h/wk) Mean ± SD**	4.07 ± 12.32		(3.20, 4.92)
Non	409	50.1	(47, 54)
1-2 hour	201	24.6	(22, 28)
≥3 hours	206	25.2	(22, 28)
**Child's household SHS exposure**			
No	409	50.1	(47, 54)
yes	407	49.8	(46, 53)

Variation in the total sample size across the various characteristics is missing.

*The percentages do not total 100 due to having been rounding off.

### Regression analysis for the avoidance behavior of SHS

Regression analysis was utilized to identify the important variables of behavior in order to avoid ETS. Ethnicity, educational level, and household monthly income were coded as dummy variables. The regression model sequences that were tested are shown in Tables 2 and 3. Here we are concerned that some of the variables, such as age, ethnicity, educational level, and household monthly income, may be associated with the respective avoidance behavior of SHS. To make sure that these variables do not explain away the entire association between the demographic variables and avoidance behavior of SHS, we put them into the model first. All potentially confounding psychological variables were put into a second and later step in the process (ex. the participant's smoking status, that of their spouse, the SHS exposure time and the child's SHS exposure time), and the variables that this study is most interested in, i.e., self-efficacy, attitude, and knowledge, were inserted into the last step.

**Table 2:** Multivariate Regression models of avoidance behavior on SHS exposure in the pre-school child's mother from 30 kindergarten schools in Hualien County, Taiwan (N = 919)

Item	β	95% CI
**Model 1**		
**Age**	.044	(-0.01, 0.12)
**Ethnicity**		
Hakka vs. Minna	-.013	(-1.04, 0.63)
Aboriginal vs. Minna	.028	(-0.43, 1.26)
Other vs. Minna	-.010	(-2.09, 1.46)
**Educational level**		
Senior high school vs. Junior high school or below	-.057	(-1.80, 0.39)
College or above vs. Junior high school or below	-.009	(-1.27, 1.06)
**Household monthly income**		
30,000 - 50,000 vs. < 30,000	.016	(-0.82, 1.23)
50,000 - 70,000 vs. < 30,000	-.016	(-1.32, 0.90)
70,000 - 90,000 vs. < 30,000	-.031	(-1.79, 0.76)
> 90,000 vs. < 30,000	-.035	(-1.98,0.70)
**Model 2**		
**Current smoker**		
Yes vs. No	-.260***	(-7.05, -4.38)
**Model 3**		
**Spouse smoked**		
Yes vs. No	-.060*	(-1.38, -0.06)
**Model 4**		
**SHS exposure time**	-.138***	(-0.07, -0.02)
**Model 5**		
**Child's SHS exposure time**	-.084*	(-0.01, -0.08)
**Model 6**		
**Self-efficacy**	.397***	(0.43, 0.59)
**Model 7**		
**Attitude**	.274***	(0.46, 0.70)
**Model 8**		
**Knowledge**	.082**	(0.15, 0.65)

* (p < 0.05), ** (p < 0.01), *** (p < 0.001)

**Model 1**: This model included socio-demographic factors.

**Model 2**: The variables included socio-demographic factors and current smoker.

**Model 3**: The variables included socio-demographic factors, current smoker, and spouse current smoker.

**Model 4**: The variables included socio-demographic factors, current smoker, spouse current smoker, and SHS exposure time.

**Model 5**: The variables included socio-demographic factors, current smoker, spouse current smoker, SHS exposure time, and child's SHS exposure time.

**Model 6**: The variables included socio-demographic factors, current smoker, spouse current smoker, SHS exposure time, child's SHS exposure time, and self-efficacy.

**Model 7**: The variables included socio-demographic factors, current smoker, spouse current smoker, SHS exposure time, child's SHS exposure time, self-efficacy, and attitude.

**Model 8**: The variables included socio-demographic factors, current smoker, spouse current smoker, SHS exposure time, child's SHS exposure time, self-efficacy, attitude, and knowledge.

**Table 3:** Multivariate Regression models of avoidance behavior on SHS exposure in the pre-school child's mother from 30 kindergarten schools in Hualien County, Taiwan (N = 919)

Model	Mult. R	**Adjust R**^**2**^ (%)	**R**^**2 **^**change** (%)	Sig. change		
Model 1	0.29	0.68	8.2	F change =	5.70	P < 0.001
Model 2	0.51	0.25	17.8	F change =	153.01	P < 0.001
Model 3	0.52	0.26	1.5	F change =	12.85	P < 0.001
Model 4	0.54	0.28	1.8	F change =	16.47	P < 0.001
Model 5	0.55	0.28	0.7	F change =	6.11	P < 0.05
Model 6	0.71	0.49	19.9	F change =	251.01	P < 0.001
Model 7	0.75	0.55	6.2	F change =	88.89	P < 0.001
Model 8	0.75	0.56	0.7	F change =	9.66	P < 0.01

**Model 1**: This model included socio-demographic factors.

**Model 2**: The variables included socio-demographic factors and current smoker.

**Model 3**: The variables included socio-demographic factors, current smoker, and spouse current smoker.

**Model 4**: The variables included socio-demographic factors, current smoker, spouse current smoker, and SHS exposure time.

**Model 5**: The variables included socio-demographic factors, current smoker, spouse current smoker, SHS exposure time, and child's SHS exposure time.

**Model 6**: The variables included socio-demographic factors, current smoker, spouse current smoker, SHS exposure time, child's SHS exposure time, and self-efficacy.

**Model 7**: The variables included socio-demographic factors, current smoker, spouse current smoker, SHS exposure time, child's SHS exposure time, self-efficacy, and attitude.

**Model 8**: The variables included socio-demographic factors, current smoker, spouse current smoker, SHS exposure time, child's SHS exposure time, self-efficacy, attitude, and knowledge.

The results reveal: that mothers who are current smokers (β = -0.260, p < 0.001), whose spouses are current smokers (β = -0.060, p < 0.05), who have SHS exposure time (β = -0.138, p < 0.001), in regard to a child's SHS exposure time (β = -0.084, p < 0.05), and that a participant's self-efficacy (β = 0.397, p < 0.001), attitude toward avoiding SHS (β = 0.274, p < 0.001), and that their level of knowledge (β = 0.082, p < 0.01) were all found to be significantly associated with SHS avoidance behavior. These variables can explain 55.5% of the total variance in behavior as to the avoidance of SHS (Table 3). "Self-efficacy" and "current smoker" alone explained 19.9% and 17.8% of the variability in the study participants' SHS avoidance behavior. We observed that the stronger one's self-efficacy, the more positive their attitude was toward avoiding SHS; moreover, the better the participant's knowledge of SHS was, the stronger their behavior to avoid SHS was. On the other hand, when the participant and their spouse/partner was a current smoker, the longer the length of exposure time to SHS for the mother and their child was, the less strong the behavior to avoid SHS was found to be. The attitude toward avoiding SHS was observed to be significantly associated the 3^rd ^most with respect to the avoidance of SHS, explaining 6.2% of the variance (Table 3).

## Discussion

### High SHS exposure

A high SHS exposure prevalence was found for a large sample of the mothers and their pre-school children in eastern Taiwan in our study (70% and 50%, respectively). This is slightly higher than that seen in another study conducted on Taiwanese children in 2004, which concluded that there was a 44.9% SHS exposure rate[5]. The high prevalence rate of exposure to SHS among pre-school child in Taiwan was 1.99 times higher than that in the U.S. 2000 national survey (49.8% vs. 25%)[10].

Furthermore, 69.7% of the mothers in our study reported that they had been exposed to SHS, a number that is much higher than the results found in the Taiwan National Health Interview Survey in 2001, which found a 58% SHS exposure rate in similar age groups[13]. The results of this study imply that the numbers for women and children, if combined, constitute the bulk of the population that is exposed to SHS.

For more than two decades, Taiwan has worked hard to control the hazard of tobacco intake. This effort has been carried out in an in depth manner in public spaces, workplaces, schools, and homes. In order to prevent SHS and to arouse people's awareness of the dangers of SHS, this has included the banning of smoking in public places. Until January of 2009, a new tobacco control act was included with the aim of preventing second-hand smoke in public places in Taiwan. Although SHS problems have not been limited to homes and other private quarters, as of 2009 such Taiwanese governmental legislative protection has not been enforceable in individual living environments, which remain the primary source of SHS exposure for women and children [5,13].

With respect to the eastern Taiwanese population, a high rate of smoking prevalence and smoking behavior has been found. The Bureau of Health Promotion reported a 50.2% current smoking rate among male adults in eastern Taiwan, indicating a relatively high prevalence of smoking there as compared to in other areas in Taiwan [24]. Moreover, in the present context, this extraordinarily high SHS exposure rate may be due to the plausible fact that men (potential husbands and fathers) have had a high smoking rate in eastern Taiwan. Another likely reason for this finding is related to traditional gender roles in Chinese culture [25], where women tend to show humble endurance without any resistance or more significant self-protective measures in regard to things like being exposed to the SHS produced by their husbands. In this way, women and their children will become more impacted victims of the harmful effects due to SHS exposure in their home. Therefore, it is quite important to address the problem of SHS exposure among mothers and their children.

### Contributing factors associated with SHS avoidance behavior

The present study explored factors that are predicted to be significant with respect to a mother's avoidance of SHS. In this study, self-efficacy was the strongest factor associated with such behavior, explaining 19.9% of the variance. In the past, self-efficacy has been an important issue in health practice promotion research. Evidence has indicated that health practices that promote self-efficacy have a significant effect on behavior performance [26-28]. This finding is consistent with another set of studies that have revealed the relative importance of a woman's degree of self-efficacy in their degree of SHS avoidance [29]. In the present context, self-efficacy was observed to function as a better variable than attitude in regard to a mother's SHS avoidance behavior. This finding differs from that in previous studies, which have concluded that their attitude works as the better predictor [30].

From the standpoint of social cognition theory, self-efficacy functions as a mediator that promotes action when an individual applies knowledge and executes it[31]. As such, there has been considerable empirical scrutiny of such a mediating role in the adoption of healthy behaviors among people of varying health statuses, ages, and ethnic groups [32-34]. Given the coherence of such theoretical assumptions and the results of this study, it is clear that the promotion of SHS avoidance should entail means for increasing the self-efficacy and empowerment of non-smoking mother's in regard to their own health choices and those of their children.

Meanwhile, this study sheds light on the body of knowledge about the SHS association to the avoidance of SHS among mothers. The results of this study are similar to those found by Kurtz et al [35]. In addition, attitudes toward was observed to be a significant factor for the avoidance of SHS, explaining 6.2% of the total variance. This result is supported by other studies and suggests that attitudes can be a predictor of an individual's behavior [30,35,36]. Indeed, a greater degree of knowledge and a more positive attitude toward avoiding SHS is a prerequisite for successful SHS avoidance.

In this study, mothers who were current smokers were less likely to adopt SHS avoidance behavior. Being a current smoker was significantly associated with the avoidance of SHS. Mothers who were current smokers were less likely to refuse to enter an environment where SHS was present, to control passive exposure by requesting smokers to stop smoking, or to attenuate exposure when unable to fully withdraw from SHS. Since it was possible to increase their and their child's SHS exposure rates, in order to better protect the health of children, further campaigns against SHS should be developed and implemented that increase the degree of a mother's smoking cessation. Furthermore, mothers should be made aware of the necessity of adopting SHS avoidance behaviors and encouraged to make their households more smoke-free at the very least [16].

This study demonstrates that spouses who smoke had an inverse effect on and were significantly associated to the degree of avoidance behavior in regard to SHS. Similarly, prior studies have found that the odds of SHS avoidance were lower among women who had a current partner that smokes [37]. Our study indicated that among mothers more than half (51.7%) reported a partner who is a current smoker. Although the smoking prevalence rate for most Taiwanese women is low, the higher smoking prevalence among men makes SHS exposure an important risk factor for nonsmokers. The adoption and maintenance of a more smoke-free home can encourage active smokers to quit or reduce consumption [38,39] and this can be an effective strategy for decreasing children's SHS exposure[40]. The Tobacco Use Supplement of the nationally representative U.S. Current Population Survey (TUS-CPS) interviewed a longitudinal population for a study and noted that adopting a smoke-free home during the year was found to significantly increase smoking cessation and to lead to a decline in tobacco consumption for those at heavier consumption levels. These national studies establish strong evidence that smoke-free homes can influence smoking behavior and reduce exposure to indoor second-hand smoke [38]. However, the relatively high frequency of SHS exposure among the mothers and their children observed in our study may result from less effective enforcement of smoke-free policies at home and/or work.

### Limitations

This study has been found to have several limitations. Firstly, since this was a cross-sectional study, evidence of any associations should be interpreted carefully before a causal relationship can be claimed. Second, since both the avoidance behavior of SHS and other independent variables included in the analysis were based on self-reported information; such data were subject to report bias. Third, since the currently decreased acceptance of SHS in a smoke-free society may have led these mothers to under-report the child's actual prevalence of SHS exposure, a social desirability bias is an important concern when interpreting these results. Notwithstanding, since all of the participants in this survey were assured of their anonymity, the threat of social desirability bias is likely to be limited here. Fourth, our findings are potentially limited by the selection of participants on more of a convenience basis, leading the results from perhaps being as readily generalized to other populations in Taiwan.

## Conclusion

The high prevalence rate of exposure to SHS in mothers and their children is quite troubling. If such a high rate of SHS exposure continues unchecked, SHS exposure will be responsible for the increased risk of mothers and their children's health consequences as a result of tobacco exposure. Self-efficacy and current smoker avoidance in regard to SHS has reached a significant degree of predictability in terms of a mother's taking SHS avoidance action. The findings further suggest the need for a well-designed intervention program to be implemented to assist pre-school children's mothers to avoid SHS exposure hazards, more specifically, the need to strengthen such a population's knowledge, to enhance their self-efficacy levels and their positive attitude in dealing with such issues.

In conclusion, theoretically directed and empirically evidence based tobacco control intervention designed programs should be quickly and carefully provided in order to encourage mothers to prevent themselves and their family members from the hazards associated with SHS exposure.

## Competing interests

The authors declare that they have no competing interests.

## Authors' contributions

PLL and HMH conceived of this study, participated in its design, developed the measure instruments, collected the data and coordinated as well as revised the manuscript. PLL and HLH performed statistical analysis, interpretation of the data, and were involved in the drafting and revision of this manuscript. KYL, TC, WTL and CHL were involved in the drafting and revision of this manuscript. All authors read and approved the final manuscript.

## Pre-publication history

The pre-publication history for this paper can be accessed here:

http://www.biomedcentral.com/1471-2458/10/606/prepub
